# Retinal nerve fibre layer, ganglion cell layer and choroid thinning in migraine with aura

**DOI:** 10.1186/1471-2415-14-75

**Published:** 2014-05-31

**Authors:** Metin Ekinci, Erdinç Ceylan, Halil Hüseyin Çağatay, Sadullah Keleş, Nergiz Hüseyinoğlu, Burak Tanyıldız, Özgür Çakıcı, Baki Kartal

**Affiliations:** 1 Ophthalmology, Univesity of Kafkas, Kars 36100, Turkey; 2 Ophthalmology, Erzurum Training and Research Hospital Ophthalmology Clinic, Erzurum, Turkey; 3 Ophthalmology, University of Atatürk, Erzurum, Turkey; 4 Neurology, University Of Kafkas, Kars 36100, Turkey; 5 Department of Ophthalmology, Istanbul University, Istanbul Faculty of Medicine, İstanbul, Turkey; 6 Ophthalmology, University of Muğla Sıtkı Koçman, Muğla, Turkey

**Keywords:** Migraine with aura, Retinal nerve fiber layer thickness, Ganglion cell layer thickness, Choroid thickness, Optical coherence tomography

## Abstract

**Background:**

The aim of this study was to investigate the thickness of the retinal nerve fiber layer (RNFL), the ganglion cell layer (GCL), and choroid thickness (CT) in patients who have migraines, with and without aura, using spectral optical coherence tomography (OCT).

**Methods:**

Forty-five patients who had migraines without aura (Group 1), 45 patients who had migraines with aura (Group 2), and 30 healthy participants (control group) were included in the study. Spectral OCT was used to measure the RNFL, GCL and CT values for all patients.

**Results:**

The mean age of Group 1, Group 2, and the control group was 34.6 ± 4.3, 32.8 ± 4.9, and 31.8 ± 4.6 years, respectively. The mean attack frequency was 3.6/month in Group 1 and 3.7/month in Group 2. The mean age among the groups (p = 0.27) and number of attacks in migraine patients (p = 0.73) were not significantly different. There was significant thinning in the RNFL and GCL in Group 2 (p < 0.05, p < 0.001 respectively), while there were no significant differences in RNFL and GCL measurements between Group 1 and the control group (p > 0.05). All groups were significantly different from one another with respect to CT, with the most thinning observed in Group 2 (p < 0.001). When all migraine patients (without grouping) were compared with the control group, there were significant differences on all parameters: RNFL thickness, GCC thickness and CT (p < 0.05).

**Conclusions:**

RNFL and GCL were significantly thinner in the migraine patients with aura as compared with both the migraine patients without aura and the control subjects. In migraine, both with aura and without aura, patients’ choroid thinning should be considered when evaluating ophthalmological findings.

## Background

Migraine is a disease presenting with an episodic headache capable of causing significant dysfunction, together with neurological, gastrointestinal, and autonomic changes, its pathogenesis is still unclear [1,2]. There are two syndrome definitions related to migraine, namely with aura and without aura [3]. The migraine aura is a mixture of focal neurological symptoms that are seen prior to an attack, accompanying an attack, and rarely after an attack. Most auras develop within 5–20 minutes, and generally last for less than 60 minutes. They may present as visual, sensory, and motor phenomena, and sometimes may affect tongue and brain stem functions. Various vascular, neurovascular, hypoxic, cellular, hormonal, and genetic hypotheses have been debated with respect to migraine pathogenesis [4]. Importantly, vasospasm in the occipital hemisphere in patients who have migraine with aura, and the subsequent reduction in the blood flow, is a major hypothesis in the explanation of visual aura and headache. In certain patients, vasospasm has been shown to occur in the tissues outside the brain, especially in the retina layer, concurrent with a reduction in brain blood flow [5,6]. There are various studies on the effects of vascular anomalies related to vasospasm and ischemia on the retina and optic nerve head [7]. Owing to technological advances in recent years, the new-generation spectral domain OCT instruments that use improved scanning speed and special software techniques (EDI) enable the acquisition of high-resolution images OCT [8], which can perform reproducible, non-invasive *in vivo* evaluations, has come into clinical use as an imaging method for evaluating the thickness of the optic nerve head, peripapillary RNFL, GCL, and choroid layers in various neuroophthalmologic diseases [9,10].

Although some studies have investigated the RNFL thickness in patients who have migraine with and without aura by using OCT and scanning laser polarimetry [1,2,11], there are no studies on the GCL and CT in these patients. The aim of the present study was to investigate potential differences in RNFL, GCL, and CT between patients who have migraine with aura and patients who have migraine without aura.

## Methods

This observational, cross-sectional and multicenter study adhered to the tenets of the Declaration of Helsinki. It was approved by the local ethical committee (*Kafkas University, Human Ethics Committee, Meeting: 2011/5, Document No:28, Kars, Turkey*) and written informed consent was obtained from all patients before they had been recruited into the study. A total of 90 patients (30 males and 60 females with a similar mean age) who were followed in the Neurology Department; were divided into 2 groups (Group 1: Migraine without aura; Group 2: Migraine with aura). Each group consisted of 15 males and 30 females. Thirty non-smoking hospital employee participants (10 males and 20 females) were included in the control group. The right eye of each participant was included in the study. The diagnosis of migraine with and without aura was made according to the 2004 guidelines of the International Headache Society [12]. Patients’ detailed histories were recorded, and information about the frequency of migraine attacks, pain localization, age at onset, duration, and the presence of aura was obtained. The type of migraine was determined, and systemic comorbidities, presence of migraine in the family, and history of glaucoma were noted. All measurements involving migraine patients were performed during both pain- and attack-free periods.

### Patient selection

One hundred and thirty-seven migraine patients were evaluated. Volunteers who had full vision (1.0 with or without correction) and who had normal ocular findings were included in the study. Forty-seven patients with myopia, central serous chorioretinopathy, retinitis pigmentosa, angioid streaks, primary angle-closure glaucoma, choroidal neovascularization, and those who had previous eye surgery for disorders such as scleral buckling, diabetes mellitus and chronic hypertension (untreated or on Ht medication), patients who had been diagnosed with Multiple Sclerosis, Parkinson’s disease, or Alzheimer’s disease and patients who smoked tobacco were excluded from the study.

### Ophthalmologic examination

**Figure 1:**
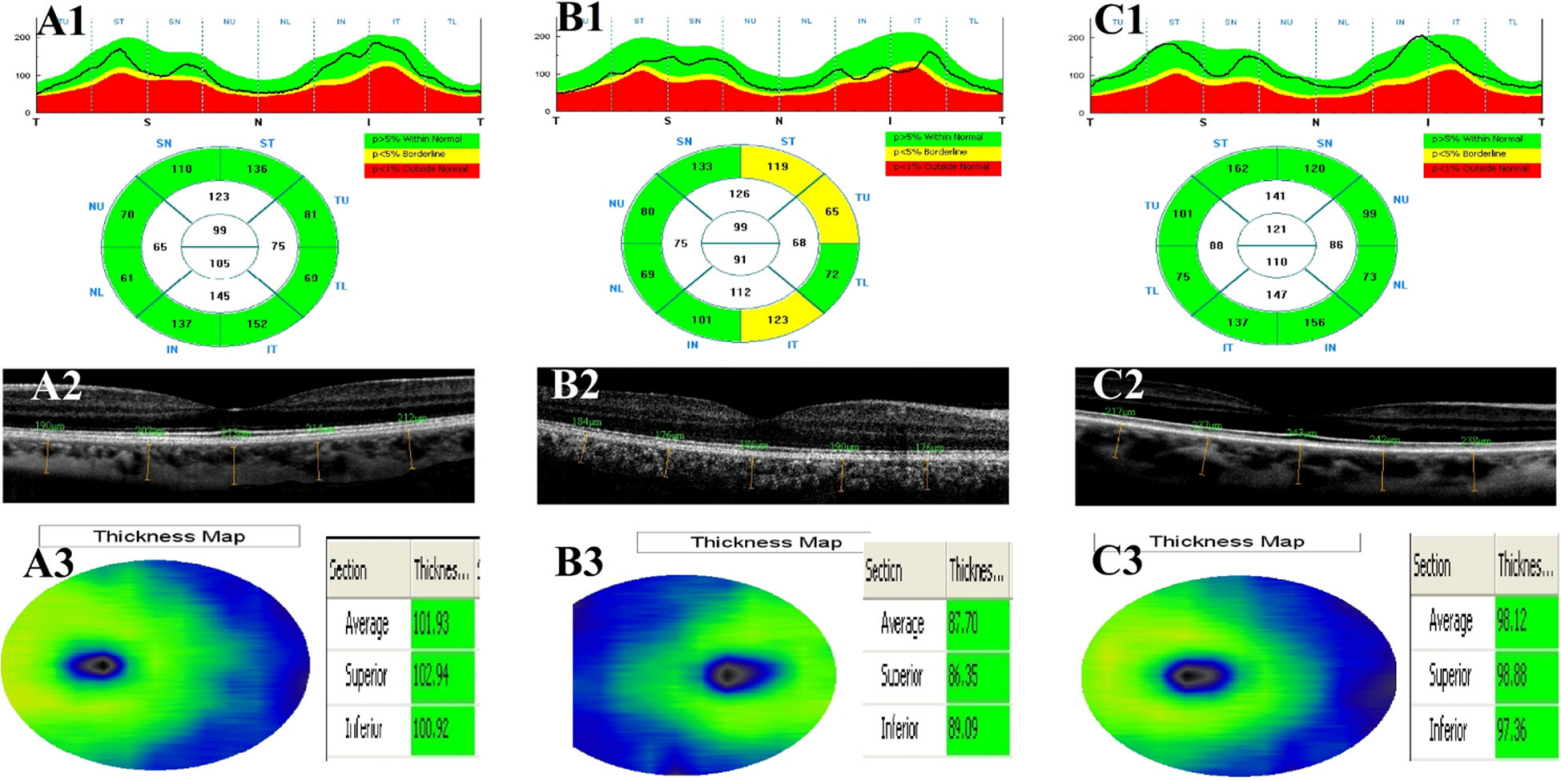
**RNFL, choroid and ganglion cell layer thickness measurments using optical coherence tomography.** A1, A2, A3: RNFL, choroid and ganglion cell layer thickness measurment of a patient in group 1. B1, B2, B3: RNFL, choroid and ganglion cell layer thickness measurment of a patient in group 2. C1, C2, C3: RNFL, choroid and ganglion cell layer thickness measurment of a patient in the control group.

Full ophthalmologic evaluations, including best-correct visual acuity, slit-lamp biomicroscopy, Goldmann applanation tonometry, gonioscopy with a three mirror contact lens, and fundoscopy were performed. RNLF, GCL and CT measurements were performed using an OCT RTVue version 4.0 (Optovue®, USA), through undilated pupils (Figure 1). Only scans that reached signal strength of at least ≥6, which indicate a high quality scan, were accepted for analysis. Considering the diurnal variations in the CT of all participants, CT was measured from 5 extra-foveal points, and RNFL and GCL thickness were measured between 10 a.m. and 12 a.m. The RNFL and GCL measurements were made to cover an area with a diameter of 3.45 mm in the horizontal and vertical planes. The instrument used a retina cross line scanning pattern that included a 1024 A-scan and consisted of two 6-mm orthogonal lines. With the automatic reversal of the image, the chorioretinal interface becomes adjacent with zero delay. The retina cross line scanning method consists of a mean of 32 patterns in 16 directions, without eye tracking. CT measurements were taken perpendicularly from the outer edge of the retinal pigment epithelium to the choroid sclera boundary at the fovea, and at four more points that are located, respectively, 500 μm nasal to the fovea, 1000 μm nasal to the fovea, 500 μm temporal to the fovea, and 1000 μm temporal to the fovea. CT measurements were made by two masked physicians (EC, ME). The average of the two measurements was used for analysis; the differences between readings of the masked physicians were found to be within 10% of the mean.

### Statistical analysis

The normal distribution of the data was checked using the Kolmogorov-Smirnov test. The non-parametric Kruskal-Wallis test for continuous data was used to compare the three groups.. Non-parametric post-hoc tests with Bonferroni correction were performed on data that had reached significance with the Kruskal-Wallis Test. Comparisons between the two experimental groups were performed with a Mann Whitney *U* test. IBM SPSS for Windows Ver. 20.0 was used for the statistical analysis, and a p value <0.01 was considered significant.

## Results

The mean ages in Group 1, Group 2, and the control group were 34.6 ± 4.3 years (27–40), 32.8 ± 4.9 years (25 – 40), and 31.8 ± 4.6 years (23–40), respectively. There were no significant differences in age among the groups (p = 0.27). The mean time required for migraine diagnosis in Group 1 and Group 2 was 88.8 ± 12.5 months (66–114) and 85.3 ± 11.9 months (62–110), respectively. The mean attack frequency was 3.6/month in Group 1 and 3.7/month in Group 2. There was no significant difference in mean time required for migraine diagnosis and mean attack frequency between Group 1 and Group 2 (p = 0.53, p = 0.73; respectively) (Table 1).

**Table 1:** Demographic results of the three groups

	**N**	**Average age**	**Migraine diagnosed (months)**	**Mean attack frequency**
		**Mean ± SD**	**Mean ± SD**	**per month**
**Group 1**	45	34.6 ± 4.3	88.8 ± 12.5	3.6
**Group 2**	45	32.8 ± 4.9	85.3 ± 11.9	3.7
**Control**	30	31.8 ± 4.6	————————	————————
*P*		*. p* = 0.27	*p* = 0.53	*p* = 0.73

SD: Standard Deviation. p: Significance.

All patients in Group 2 reported visual symptoms such as light flashes, light refraction, and black dots in the visual area.

When the groups were evaluated according to their median RNFL thickness, there were significant differences between Group 1 and Group 2 regarding overall, superior, and inferior faces (p = 0.02, p = 0.04, p = 0.04, respectively). No significant differences were found between Group 1 and the control group (p > 0.05 for all). On the other hand, there were significant differences between Group 2 and the control group regarding all faces (p < 0.05 for all) except for the nasal face (p = 0.10) (Table 2).

**Table 2:** Median OCT analysis results of the three groups

	**RNFL thickness (μm)**					**GCL thickness (μm)**		**CT (μm)**
	**(min-max)**					**(min-max)**		**(min-max)**
	**Overall**	**Temporal**	**Superior**	**Nasal**	**Inferior**	**Superior**	**Inferior**	
**Group 1**	112.40	89.60	130.20	75.40	140.20	97.55	96.25	191.70
	(101–117)	(76–92)	(122–136)	(69–81)	(131–145)	(93–99)	(96–102)	(174–216)
**Group 2**	103.45	81.20	122.30	72.80	133.60	93.40	96.10	168.40
	(92–107)	(72–86)	(116–128)	(63–77)	(123–138)	(88–96)	(89–98)	(154–190)
**Control**	116.20	86.30	136.40	80.60	145.30	98.90	102.40	228.10
	(107–127)	(79–96)	(122–142)	(68–88)	(133–151)	(95–101)	(96–104)	(205–243)

RNFL: Retinal nerve fiber layer. GCL: Ganglion cell layer. CT: Choroid thickness. SD: Standard Deviation.

***p***: Significance.

**RNFL**: Group 2 < Group 1 = Group Control.

**GCL**: Group 2 < Group 1 = Group Control.

**CT**: Group 2 < Group 1 < Group Control.

When the groups were evaluated according to their median GCC thickness, there were significant differences between Group 1 and Group 2 regarding both superior and inferior faces (p = 0.02, p = 0.003, respectively). While there were no significant differences between Group 1 and the control group (p = 0.36, p = 0.75; respectively), there were significant differences between Group 2 and the control group regarding both faces (p < 0.001 for both) (Table 2).

There were significant differences among all groups with respect to median CT values (p < 0.001 for all) (Table 2).

When comparing all migraine patients (without grouping) to the control group, we found significant differences on all parameters; RNFL thickness in overall (p = 0.001), temporal (p = 0.001), superior (p = 0.001), nasal (p = 0.001), and inferior (p = 0.001) faces; GCC thickness in the superior (p = 0.002) and inferior (p = 0.003) faces; and CT (p < 0.001) (Table 3).

**Table 3:** Median OCT analysis results of the migraine and control groups

	**RNFL thickness (μm)**					**GCL thickness (μm)**		**CT (μm)**
	**(min-max)**					**(min-max)**		**(min-max)**
	**Overall**	**Temporal**	**Superior**	**Nasal**	**Inferior**	**Superior**	**Inferior**	
**Migraine**								
**Patients**	103.50	83.40	125.60	71.4	134.80	92.50	94.60	174.40
(n = 90)	(92–117)	(72–92)	(116–136)	(63–81)	(123–145)	(88–99)	(89–102)	(154–216)
**Control**	116.20	86.30	136.40	80.60	145.30	98.90	102.40	228.10
(n = 30)	(107–127)	(79–96)	(122–142)	(68–88)	(133–151)	(95–101)	(96–104)	(205–243)
***p***	*p* = 0.001	*p* = 0.001	*p* = 0.001	*p* = 0.001	*p* = 0.001	*p* = 0.002	*p* = 0.003	*p* < 0.001

RNFL: Retinal nerve fiber layer. GCL: Ganglion cell layer. CT: Choroid thickness. SD: Standard Deviation. p: Significance.

## Discussion

We investigated the correlations among RNLF, GCL, and CT values and migraine. When we collapsed the two migraine conditions and compared those patients to the control group, we found significant thinning of the RNLF, GCL and choroid in the migraine patients. When the patients were evaluated as subgroups of migraine with aura, without aura and a control group, there was a significant thinning of the RNLF, GCL and choroid in migraine patients with aura, but no significant differences in RNLF and GCL measurements between migraine patients without aura and healthy subjects.

As far as we know, this is the first study to investigate the relationship between GCL, CT values and migraine.

Migraine is a chronic, progressive neurological disorder with unknown etiology, and progresses with episodic headache attacks [3]. The most common known event in migraine pathogenesis is vascular dysregulation (vasospastic diathesis) [13]. The neurovascular system is the most affected system in this pathology. Studies have detected an increased risk for ischemic stroke in patients who had migraine with aura [14,15]. Vasospasm emerging prior to or during the pain has been considered to occur concurrently in tissues located outside the brain, and local infarctions, by extension, have been considered to lead to histopathological and functional disorders at the tissue level. Various studies related to the vascular theory have been carried out in recent years to demonstrate changes in the retinal layer [5,6]. Killer et al. demonstrated a reduction in blood flow in the lower temporal artery in a patient who had a visual area defect in the left eye during a migraine attack [16]. According to another hypothesis, the vasoconstrictive metabolites that join the systemic circulation during a migraine attack affect the retina as a result of retrobulbar flow, thus reducing local blood flow [1]. Kara et al. used colored Doppler ultrasonography and found a reduction in blood flow at the level of the central retinal artery and posterior ciliary artery in migraine patients, compared to healthy individuals [17].

Tan et al. [2] measured RNFL thickness in 15 patients who had migraine with aura and 24 patients who had migraine without aura using a scanning laser polarimetry instrument, and detected that there was no reduction in RNFL thickness in migraine patients compared to healthy individuals. On the other hand, although the method for measuring the retinal nerve fiber layer (RNFL) was different (Scanning Laser Polarimetry), Martinez et al.’s study results suggest a significantly difference between the migraine patients with aura and those without aura [11]. Another of their another studies, Martinez et al. [1] used OCT to measure the RNFL, and determined that the mean RNFL thickness in migraine patients was similar to that of healthy individuals, while only the thickness of the temporal quadrant RNFL was reduced in patients compared to the control group. Gippono et al. [18] found no difference in the foveal thickness and macular volume in female migraine patients compared to healthy women, but determined that there was a significant thinning in the RNFL thickness in the upper quadrant in the female migraine patients. In the present study, the thinning of the RNFL and GCL was detected only in patients who had migraine with aura. It is possible that Tan et al. obtained different results because they used a scanning laser polarimetry instrument to measure the RNFL thickness. On the other hand, Gipponi et al. may have obtained different results because they did not classify the patients according to the presence of aura. Several studies have emphasized that cerebral hypoperfusion, which occurs most commonly in the posterior region of one hemisphere during the aura period [1], and the risk of ischemic stroke, cardiac diseases, intracerebral hemorrhage and mortality, increases in migraine patients with aura [15]; the pathogenic mechanism might be endothelial and vascular smooth muscle dysfunction and hypercoagulability [19]. Different studies have shown that neurodegenerative changes occur secondary to the subclinical ischemic lesions that form after hypoperfusion [14,15] . Moreover, some studies have reported a thinning in RNFL measurements in neurodegenerative diseases such as multiple sclerosis, Alzheimer’s disease, and Parkinson’s disease [20-22]. In light of our findings in the present study, we propose that the thinning of the RNFL is a secondary event to the neurodegenerative changes in the central nervous system after ischemia in the migraine patients with aura.

The choroid is the vascular compartment of the eye. It supplies oxygen and nutrients to the outer retina. The major blood supply to the retina is the choroid, especially in darkness, where 90% of the oxygen comes from choroidal circulation [23]. In the literature, Bourke et al. defined the correlation between untreated systemic hypertension and choroidopathy [24], Regatieri et al. defined CT in diabetic retinopathy and suggested that CT was associated with retinal tissue hypoxia [25], and Steigerwalt et al. and Sızmaz et al. reported a decrease in CT in patients who smoked cigarettes, due to the increase in vascular resistance of the vessels [26,27]. According to the before-mentioned studies, migraine is known to be a neurovascular disease, and is known to reduce the blood flow at the level of the central retinal artery and posterior ciliary artery; the thinning of the choroid layer is an expected clinical outcome in migraine patients. Similarly, we found choroidal thinning in patients who had migraine both with aura and without aura.

Our study had some limitations. First, we did not grade migraine severity as was done in the Martinez et al. study. Second, we did not have access to brain MRIs to support our theory and to show ischemic changes in migraine with aura, because ethical committee disallowed this procedure.

Overall, a structurally and functionally normal retinal and choroidal vasculature is essential for the function of the retina: abnormal choroidal blood volume and/or compromised flow can result in photoreceptor dysfunction and death [28]. Changes in retinal and choroidal blood flow may be important in retinal and choroideal pathologies.

## Conclusion

We report the correlations among RNLF, GCL, and CT values and migraine with aura. Further investigation with a larger number of cases might provide more information about the relationship between retinal-choroidal pathologies and migraine with aura.

## Competing interests

The authors declare that they have no competing interests.

## Authors’ contributions

Conceived and designed the study: ME, EC. Acquisition of data: HHÇ, NH. Analysis and interpretation of data: ME, EC, SK. Drafting the manuscript: ME, EC, BT, ÖÇ. Revising the manuscript critically for important intellectual content: BK. All authors read and approved the final manuscript.

## Pre-publication history

The pre-publication history for this paper can be accessed here:

http://www.biomedcentral.com/1471-2415/14/75/prepub
